# Typhus Group Rickettsiae Antibodies in Rural Mexico

**DOI:** 10.3201/eid1003.030438

**Published:** 2004-03

**Authors:** Virginia E. Alcantara, Esperanza G. Gallardo, Chao Hong, David H. Walker

**Affiliations:** * WHO Collaborating Center for Tropical Diseases, University of Texas Medical Branch, Galveston Texas, USA; † Instituto de Salud del Estado de Mexico, Toluca, Mexico

**Keywords:** *Rickettsia prowazekii*, louse-borne typhus, Brill-Zinsser disease

**To the Editor:** In 2002, the risk of transmission of epidemic typhus in the state of Mexico was assessed by analyzing serum specimens from 393 residents of previous typhogenic areas for immunoglobulin (Ig) G antibodies against *Rickettsia prowazekii*. Louseborne typhus has been a historic scourge in Mexico. In 1576, in a population of 9 million, 2 million deaths were attributed to epidemic typhus (*1*). These illnesses primarily affected indigenous peoples, who called the illness *cocolixtle* and *matlazahuatl* (*2*).

In 1951 a national campaign against louseborne typhus was begun by using newly developed technologic approaches, antibiotics, and insecticides, resulting in decreases in the incidence and case-fatality rate. In 1951 >1,000 cases and 737 deaths caused by epidemic typhus were reported in 18 states, and 6,781 localities were identified as at risk (*3*). By 1965, only 36 cases and no deaths were reported from 12 states with 4,841 localities at risk. Most cases occurred during the cold months of November–April. One third of cases occurred in persons 19–29 years of age with nearly 40% of the deaths in patients aged 15–44 years. In 1979, 10 years had passed without any cases of epidemic typhus reported in Mexico. In the 1980s, three outbreaks of typhus occurred in rural communities, two in Chiapas and one in the state of Mexico (*4*).

In the state of Mexico, during the period 1893–1907, 7,353 epidemic typhus deaths were reported (annual mortality rate, 52.4/100,000 population); from 1939 to 1943, 1,220 cases were reported with 707 deaths (annual mortality rate, 12.1/100,000 population); and from 1959 to 1963, 64 cases were reported with 14 deaths (annual mortality rate, 0.1/100,000 population) (*3*). In 1967, Atlacomulco, a county in the state of Mexico that had been free of typhus for 5 years, experienced an outbreak of louseborne typhus associated with a case of Brill-Zinsser disease in a 76-year-old man who had a history of epidemic typhus. Forty cases were diagnosed and one death occurred (*3*). The last outbreak in the state of Mexico occurred in 1983 in San Juan Cote in San Felipe del Progreso County, with 22 ill persons and one death (*4*). Since then, a public health program against *Pediculus humanus corporis* has been conducted in five counties with epidemiologic surveillance for cases of reactivation of latent infection. At the beginning of the 1980s, the rate of infestation with *P. humanus corporis* (*mazahua*) in the indigenous population of the state of Mexico was 100%; in 1988, 58%; and in 1990, 15%. In 1999, indices of infestation between 5% and 12% were detected in this population (*5*).

In 2002, personnel from the office of the Secretary of Health of the State of Mexico evaluated the risk to the population who lived in previously typhogenic areas to measure the impact of their programs (*5*). In a cross-sectional study, 393 human serum specimens were analyzed by immunofluorescence assay (IFA) for IgG antibodies against *R. prowazekii,* and a titer of 64 or higher was considered positive (*6*). Antibodies against *R. prowazekii* were detected in 74 serum samples (seropositivity, 18.8%; 26% for males and 18% for females). The prevalence of antibodies to *R. prowazekii* increased with age; 1–14 years of age (seropositivity 0%), 15–24 years (14%), 25–44 years (17%), 45–64 years (24%), and >65 years (48%). Thirty-three (45%) of the serum specimens had a titer of 64, 25 (34%) had a titer of 128, and 16 (22%) had a higher titer. All eight serum specimens with a titer of >512 were from persons >45 years of age.

The high seroprevalence suggests that this population had extensive exposure to the agent of typhus and its louse vector in the past. The finding of two subjects aged >65 years with a titer of 1,024 and four subjects aged >45 years with a titer of 512 suggests reactivation of latent *R. prowazekii* with a resulting boost in their antibody titers. These possible cases of Brill-Zinsser disease were likely not severely ill and recovered either with antimicrobial treatment that was effective against *R. prowazekii* or by immune control of the infection without a specific diagnosis.

That IgG antibodies against *R. prowazekii* are absent in young persons suggests that this rickettsia has not been circulating in this population during recent years. The high seroprevalence suggests a human reservoir of latent *R. prowazekii* in this population. The presence of human body lice in this population indicates that there is risk of spreading *R. prowazekii* from an index patient with Brill-Zinsser disease to persons in contact with the patient.

Although the general lack of attention to *R. prowazekii* by scientists, physicians, and public health agencies would lead one to believe that typhus has been eliminated as a public health concern, the recent occurrence of a large epidemic in Burundi (*7*), infected lice in Rwanda, an outbreak in Russia, a documented case originating in Algeria, and outbreaks every year in Andean Peru (*8*) indicate that global attention should be directed to surveillance, risk assessment, diagnostic capability, and planning for rapid epidemic control to avoid establishing a large reservoir of latent infection for future epidemics originating from recrudescent typhus in louse-infested populations. Typhus likely poses a similar threat in other parts of the world including the Himalayas, Andes, Afghanistan, and highlands of Africa. Even in industrialized countries, the diagnosis of typhus is likely to be delayed or missed. The potential threat of bioterrorist-disseminated, aerosol-transmitted typhus emphasizes that enhanced attention to and knowledge of typhus are needed throughout the world (*9*). The requirement concerns not only physician awareness but also wide availability and application of the most appropriate diagnostic laboratory methods.
